# Effectiveness of 0.2% Hyaluronic Acid on Clinical, Biomolecular and Microbiological Parameters in Type 2 Diabetes Mellitus Patients with Periodontitis

**DOI:** 10.1055/s-0044-1782188

**Published:** 2024-07-29

**Authors:** Anggun Alfreda Devina, Felita Clarissa Halim, Meivi Meivi, Sri Lelyati C. Masulili, Ette Soraya Shahnaz Tadjoedin, Robert Lessang, Adityo Widaryono, Boy M. Bachtiar, Benso Sulijaya, Fatimah Maria Tadjoedin, Natalina Haerani, Nadhia Anindhita Harsas, Astrid Diana Bakker

**Affiliations:** 1 Periodontology Specialist Program, Department of Periodontology, Faculty of Dentistry, Universitas Indonesia, Jakarta, Indonesia; 2 Department of Periodontology, Faculty of Dentistry, Universitas Indonesia, Jakarta, Indonesia; 3 Department of Oral Biology, Faculty of Dentistry, Universitas Indonesia, Jakarta, Indonesia; 4 Oral Cell Biology Department, Academic Centre for Dentistry Amsterdam, ACTA, Amsterdam, the Netherlands

**Keywords:** diabetes mellitus, hyaluronic acid, Porphyromonas gingivalis, Fusobacterium nucleatum, interleukin-1β, interleukin-10

## Abstract

**Objective** This double-blind randomized clinical trial assessed the effectiveness of 0.2% hyaluronic acid (HA) gel as an adjunct to scaling and root planning (SRP) in patients with periodontitis and type 2 diabetes mellitus (DM), focusing on changes in clinical periodontal parameters, the expression of inflammatory mediators, and oral pathogens.

**Materials and Methods** The randomized clinical trial involved 36 participants, 18 DM patients, and 18 healthy patients. The participants in each group were randomly assigned to receive placebo or HA gel after SRP. Gingival crevicular fluid and subgingival plaque samples were taken before treatment and at 4-week follow-up. Clinical parameters, interleukin-1β (IL-1β) and IL-10 levels, and proportions of *Porphyromonas gingivalis (Pg)* and *Fusobacterium nucleatum (Fn)* were evaluated at baseline and follow-up.

**Statistical Analysis** Paired *t* -test (parametric data) or Wilcoxon signed-rank test (nonparametric data) was used for intragroup comparison between baseline and follow-up, and comparisons between groups one-way analysis of variance test (parametric data) or Kruskal–Wallis test (nonparametric data).

**Results** At 4 weeks, most of the groups showed statistically significant decreases ( *p* ≤ 0.05) in various clinical and biomolecular parameters. However, there were exceptions: the pocket probing depth (PPD) and clinical attachment loss (CAL) parameter did not significantly decrease for the placebo ( *p* > 0.05) non-DM group, and the IL-10 parameter in the DM HA gel group ( *p* = 0.108). Regarding bacterial proportions, the non-DM and DM placebo group exhibited significant test results for *Pg* after 4 weeks ( *p* ≤ 0.05). In the case of *Fn* bacteria proportions, they decreased in all groups, but these results were not statistically significant ( *p* *≥* 0.05). An intergroup analysis revealed no significant differences ( *p* ≤ 0.05) for bleeding on probing (BOP), PPD, and both proinflammatory and anti-inflammatory cytokines. Only clinical attachment loss (CAL) exhibited a statistically significant intergroup difference 0.042.

**Conclusion** The use of 0.2% HA gel into periodontal pockets alongside SRP, for both diabetic and healthy individuals, showed no statistically significant variances in clinical, biomolecular, and microbiological measures.

## Introduction

Periodontitis is an inflammatory condition that affects the supportive tissues of the teeth, including the gingiva, periodontal ligaments, and alveolar bone. It results in progressive tissue damage characterized by the loss of periodontal attachment, alveolar bone resorption, tooth mobility, tooth loss, and can have an impact on a person's chewing function and quality of life. 1 This occurs due to an imbalance in the interaction between oral pathogens and the host. Epidemiologically and mechanistically, this disease is associated with systemic conditions, one of which is diabetes mellitus (DM). 2 3

DM is a common metabolic disorder resulting from abnormalities in insulin secretion, insulin function, or a combination of both. Type 2 DM is characterized by ineffective insulin utilization by the body and accounts for 90% of diabetes cases worldwide. 4 Long-term complications of DM may include cardiovascular disease, diabetic retinopathy, and kidney failure. 5 There is a close and interrelated relationship between periodontal disease and DM. Both are chronic inflammatory conditions with a high prevalence. 6 Epidemiological studies show that diabetes increases the risk and severity of periodontal disease. 7 Patients with DM are at greater risk, two to three times more likely to experience periodontal issues. 6

Poor glycemic control in diabetic patients can increase gingival inflammation and the severity of periodontitis. Conversely, periodontitis can also raise blood glucose levels. 6 Elevated blood sugar serves as a breeding ground for bacteria. 8 Patients with DM and periodontitis are associated with immune response disturbances, changes in vascularization, increased glucose accumulation in periodontal tissues, heightened proteolysis and osteolysis of periodontal structures, elevated concentrations of proinflammatory cytokines, reduced collagen structure regeneration, and the development of oral pathogens. 6 Hyperglycemia in patients with DM induces systemic inflammation characterized by the release of proinflammatory cytokines such as interleukin-1β (IL-1β), interleukin-10 (IL-10), and prostaglandin. It also increases the secretion of matrix metalloproteinases, leading to the destruction of periodontal tissues. 6 7 8 9 10 11

IL-1β is one of the factors known to stimulate bone resorption and proteinase secretion, and it may be involved in attachment loss and bone resorption, which are characteristics of periodontitis. Clinical studies in both humans and experimental animals have found increased levels of IL-1β, tumor necrosis factor-alpha (TNF-α), and IL-6 in uncontrolled diabetic patients, can lead to a worsening of the periodontal status. 10

IL-10 is a potent anti-inflammatory cytokine that plays a strong role in suppressing inflammation and immune proliferative responses. Gene polymorphisms in the IL-10 promoter have been associated with the development of periodontal disease. IL-10 levels are lower in individuals with DM, as evidenced by hemoglobin A1c (HbA1c) levels, and are inversely related to individuals who are healthy, where IL-10 levels are typically the highest. This suggests that IL-10 may have a protective role in maintaining a balanced immune response and mitigating inflammation in health. 6

According to studies, the dominant pathogenic bacteria causing chronic periodontitis is *Porphyromonas gingivalis* ( *Pg)* . 13 This bacterium is anaerobic and is more commonly found in deep periodontal pockets. *Porphyromonas gingivalis* is known to induce periodontal inflammation and is frequently found in cases of periodontitis associated with DM. 12 13 The increase in glucose levels in the gingival crevicular fluid (GCF) of diabetic patients can provide a source of nutrition and a breeding ground for subgingival microorganisms, altering the composition and quantity of specific species within the biofilm. 15

*Fusobacterium nucleatum* ( *Fn* ), which is part of the orange complex, plays a role in bridging the colonization of early bacteria and the core group that supports the colonization of red complex bacteria. 13 The condition of patients with periodontitis and DM is associated with the quantity of pathogenic bacteria that stimulate the initiation of an inflammatory response in the supporting tissues of the teeth. Species such as *Pg* and *Fn* act as triggers for the immune response to inflammation. 14

The limitations of conventional scaling and root planning (SRP) include restricted access due to factors like anatomical variations of teeth and deep periodontal pockets. 15 Consequently, achieving complete elimination of microorganisms becomes challenging, and bacterial reservoirs persist in these areas, sustaining microbial activity. Therefore, adjunctive therapy has been studied in order to overcome these challenges. 16 Hyaluronic acid (HA) is a polymer of glucuronic acid alternating with N-acetylglucosamine. 17 HA plays a role in forming the extracellular matrix structure for cell migration and proliferation. 18 Another role of HA is to maintain structural integrity and homeostasis in body tissues. It also activates metalloproteinase inhibitors, which accelerate the wound healing process and have the potential to reduce tissue trauma where stimulation in the wound healing process can occur. 19

Another advantage of HA is that it is a biocompatible compound, easy to use, and can be tailored to various anatomical morphologies. 20 The important role of HA has been widely utilized in the field of dentistry. One of the benefits of HA is its use in nonsurgical periodontal therapy as a drug delivery system. Its effectiveness is also evident in surgical procedures such as gingival recession coverage, papilla tissue regeneration, root conditioning, 21 and infrabony defects. 20 In fact, HA has been proven effective in treating implant-related inflammation. 21 HA can also provide anti-inflammatory, antiedema, and anti-bacterial effects for individuals with gingivitis and periodontitis. 18

In patients with DM and periodontitis, there are often impediments to the healing process. Despite the proven ability of HA to promote wound healing and reduce inflammation, the outcomes regarding the effectiveness of HA administration still display inconsistency. Currently, there is a lack of studies comprehensively assessing clinical, biomolecular, and microbiological parameters following SRP with HA gel as adjunctive therapy in both diabetic and non-diabetic patients. The aim of this research is to assess the effectiveness of 0.2% HA gel on periodontal parameter, quantity of *Pg* and *Fn* , and the levels of proinflammatory cytokine, IL-1β, and anti-inflammatory cytokine, IL-10, in DM patients with periodontitis.

## Materials and Methods

### Study Design and Patient Selection

This study is a parallel, double-blind, randomized clinical trial with 1:1 allocation ratio carried out at the Clinic of the Periodontology Department, Universitas Indonesia between March 2023 and June 2023. The study protocol was accepted by the Ethical Commission for Dental Research (KPEKG), Faculty of Dentistry, Universitas Indonesia under the code 130 /Ethical Approval /FKGUI/XII/2022 with protocol number 090660722 and is already recognized by The Strategic Initiative for Developing Capacity in Ethical Review (SIDCER)—Forum for Ethical Review Committees in Asia and the Western Pacific (FERCAP). The trial registration number is ISRCTN49272905. All participating patients were thoroughly briefed on the protocol and provided written consent before their involvement in the study.

Before the study, sample size was calculated using G*Power analysis program. 22 The sample size was determined based on pocket probing depth (PPD) as the primary outcome measure assuming normality. At least 16 subjects in each group (total 32 patients) were required for the detection of significant difference ( *p* ≤0.05) at 80% power test. The sample estimate experiencing dropout is 20%, resulting in a sample size of 20 individuals per group.

The inclusion criteria for this study include both male and female participants within the age range of 40 to 65 years. Participants must be diagnosed with periodontitis according to European Federation of Periodontology (EFP) 2017 23 Interdental clinical attachment loss (CAL) is detectable at more than or equal to 2 nonadjacent teeth, or buccal CAL of more than or equal to 3mm with pocketing of more than 3mm is detectable at more than or equal to 2 teeth. Samples are selectively obtained from a single site with PPD within the range of 4 to 6 mm. Participants must have undergone an HbA1c test in the last month. Those with HbA1c levels below 6.5% will be categorized into the non-DM group, whereas those with results above 6.5% will be classified into the DM group. Additionally, DM participants are required to obtain medical clearance for SRP treatment. Furthermore, they are required to demonstrate their willingness to actively engage in the research and commit to signing an informed consent form.

The exclusion criteria are factors that may impact the research outcomes such as (1) participants who regularly use medications known to affect microbes, including antibiotics, anti-inflammatory drugs, immunosuppressive drugs, bisphosphonates, and blood thinners, are not eligible for inclusion; (2) individuals with bleeding disorders, mental disorders, and acute infections are excluded from the study; (3) individuals who have received periodontal therapy within the past six months; (4) individuals that are allergic to HA gel; (5) pregnant or breastfeeding women. These exclusion criteria are implemented to ensure the accuracy and integrity of the research findings. During the course of the study, if a patient experiences a condition requiring antibiotic treatment, they will be dropped from the study as antibiotics can introduce bias into the research results. Additionally, individuals who fail to attend the specified follow-up appointment will also be excluded from the study.

### Periodontal Examination

Clinical examination of periodontal parameters was carried out for included patients by one calibrated examiner. For each patient, periodontal parameters were documented in six sites for each tooth: mid-buccal, mid-lingual, mesio-buccal, mesio-lingual, disto-buccal, and disto-lingual. Bleeding on probing (BOP), PPD, and CAL utilizing UNC-15 (Hu-Friedy, Chicago, IL, USA) periodontal probe at baseline before starting nonsurgical periodontal therapy and later during follow-up at 4 weeks intervals.

### Periodontal Therapy and Randomization

The initial visit involves the assessment of all clinical parameters, accompanied by the collection of GCF and subgingival plaque specimens, as well as SRP and gel application. Prior to this, thorough intraexaminer and interexaminer calibration practices have been carried out to ensure precision and consistency in the assessment process.

The evaluation of all clinical parameters, along with the collection of GCF and subgingival plaque specimens, will be executed by one operator (AAD) on the predesignated teeth using three to four paper points of size 25, inserted into the periodontal pocket for a duration of 30 seconds. Subsequently, all subjects underwent a full-mouth supragingival and subgingival SRP procedure, administered by a single operator (MM) using an ultrasonic device.

The test and control groups will be randomly assigned to receive either a placebo gel (control group) or 0.2% HA gel (test group). The randomized allocation was conducted by the Faculty of Pharmacy, Universitas Indonesia, which prepared the 0.2% HA gel and placebo gel in identical coded formulations, in the form of one cc syringes. One operator (FCH) will administer the gel to both the DM and non-DM groups. The operator will not be aware of the substance given to the subjects.

Participants were provided with comprehensive instructions on oral hygiene practices and optimal brushing techniques to adhere to throughout the period from baseline to follow-up. During the 4-week follow-up session, a thorough reassessment of all periodontal parameters was carried out, together with the collection of GCF and subgingival plaque samples. Adhering to standard procedures, a protocol for monitoring adverse events was implemented, ensuring the maintenance of safety and ethical standards throughout the research.

### Gingival Crevicular Fluid and Subgingival Plaque Sampling

GCF and subgingival plaque samples were obtained at baseline and during 4-week follow-up after clinical examination from selected periodontal pockets. Sample sites were gently dried using an air syringe and isolated with cotton rolls. Subsequently, sterile paper points, sized 25, were inserted inside the periodontal pockets for a duration of 30 seconds. Paper points contaminated with blood and saliva were disposed of, while others were placed inside the Eppendorf tube filled with PBS solution. A total of five paper points were collected from each sample site and stored at −20°C for final analysis.

### Measurement of IL-1β and IL-10

GCF samples for IL-1β (Human interleukin-1β, ELISA Kit, Bioenzy, Indonesia) and IL-10 (Human interleukin-10, ELISA Kit, Bioenzy, Indonesia) were assessed using enzyme linked immunosorbent assay (ELISA). The concentrations of IL-1β and IL-10 in the samples were determined using a standard curve. Add 50 µL of standard to a standard well that has not been biotinylated, and 40 µL of sample to the sample wells. To enhance the accuracy of ELISA testing, we implemented a precautionary measure by replicating the samples. Continue by adding biotinylated antibody to sample wells, followed by 50 µl streptavidin-HRP to sample and standard wells, and mixing well. Apply a sealant to the plate. Incubate at 37°C for 60 minutes. Dissolve the sealant and wash the plate with a wash buffer five times. For each wash, soak wells in at least 0.35 mL wash buffer approximately 30 seconds until 1 minute. Add 50 µL of substrate solution A into each well, followed by 50 µL of substrate solution B. Incubate the plate in the dark for 10 minutes at 37°C with a new sealer. Add 50 µL of the stop solution into each well; the blue color will transform into yellow rapidly. Within 10 minutes of applying the stop solution, immediately apply a microplate reader set to 450 nm to identify the optical density measurement for each well. IL-1β and IL-10 concentrations were represented in pg/mL. 24 To enhance the transparency of the methodology, testing was conducted in accordance with standard procedures with a detailed protocol. Similar to data collection, instrument calibration, sample handling and storage methods, and analysis techniques adhered to the specified qualifications.

### Measurement of *Pg* and *Fn* Using Quantitative PCR (qPCR)

Bacterial DNA was isolated from the subgingival plaque samples by following the manufacturer's recommendations for the GENEzol reagent (General, Ltd, New Taipei City, Taiwan). In order to minimize potential errors in real-time polymerase chain reaction (PCR) testing, a precautionary measure was taken by duplicating samples. Subsequently, DNA synthesis was performed using a reverse transcription kit from Applied Biosystems. The resulting DNA underwent triplicate amplification on an ABI StepOnePlus real-time PCR system, employing the SYBR Green PCR Master Mix (Applied Biosystems).

The real-time PCR conditions consisted of an initial denaturation at 95°C for 2 minutes, followed by 40 cycles of denaturation at 95°C for 5 seconds, annealing at 60°C for 30 seconds, and extension at 72°C for 30 seconds. Finally, there was a concluding extension at 95°C for 1 minute, followed by annealing at 62°C for 1 minute, and a final step at 95°C for 15 seconds. The findings of this study reveal a singular melting curve, indicative of a single DNA product generated during PCR. The specific primer sequences utilized in this study, as shown in Table 1 , for *Pg* , *Fn* , and total bacteria were taken from a study conducted by Kugaji et al, 25 Miranda et al, 26 and Johnson et al. 27 This observation holds significance as it aids in assessing the specificity and successful amplification of a particular genetic target. The obtained data were subjected to analysis employing the 2 ^−ΔΔCT^ method. 28 29

**Table 1:** Primers used in this study

Name	Sequences	Reference
Porphyromonas *gingivalis*	*Forward primer:* 5′-AGG CAG CTT GCC ATA CTG CG-3′ *Reverse primer:* 5′-ACT GTT AGC AAC TAC CGA TGT-3′	Kugaji et al 2019 25
*Fusobacterium nucleatum*	*Forward primer:* 5′-GCG CGT CTA GGT GGT TAT *Reverse primer:* 3′-TAG TTC CGC TTA CCT CTC CAG	Miranda et al 2017 26
Total bacteria	*Forward primer:* 5′ TTA AAC TCA AAG GAA TTG ACG G 3′ *Reverse primer:* 5′CTC ACG ACA CGA GCT GAC GAC 3′	Johnson et al 2019 27

The abundance of each bacterium was assessed through the application of the 2 ^−ΔΔCt^ method. ΔCt was determined as the variance between the cycle threshold (Ct) value obtained using primers specific to each bacterium and the Ct value derived from primers targeting total bacteria Following this, ΔΔCt represented the distinction between the ΔCt values of the patients and those of the control subjects. The resulting 2 ^−ΔΔCt^ value signifies the changes in bacterial proportions within the patient sample as compared to the control subjects. The control exhibited a 2 ^−ΔΔCt^ value of 1. 28

### Statistical Analysis

Each clinical, microbiological, and immunological parameter was computed per subject and then averaged across patients in both groups. Changes in BOP, PPD and CAL, proportions of microbial complexes, and levels of cytokines over time were examined in subsets of sites according to initial PPD of 4 to 6 mm. Normality determination was checked using the Shapiro–Wilk test. For comparisons of BOP, PPD, CAL, proportions of microbial complexes (2 ^−ΔΔCt^ ) and levels of cytokines between baseline and follow-up, the analyses were performed by means of a Paired *t* -test (parametric data) or Wilcoxon signed-rank test (nonparametric data). For comparisons between groups one-way analysis of variance (ANOVA) test (parametric data) or Kruskal–Wallis test (nonparametric data) was performed for the delta calculation of baseline and follow-up. *p* -Value less than or equal to 0.05 was considered statistically significant. For multiple comparisons, the Bonferroni posthoc adjustment test of Tukey was performed when the results of the one-way ANOVA test (for parametric data) were statistically significant. Additionally, the posthoc Dunn-Bonferroni test was conducted when the Kruskal–Wallis test (for nonparametric data) yielded statistically significant results. Statistical calculations were conducted using SPSS 26 (Statistical Package for the Social Sciences) for Windows.

## Results

**Fig. 1:**
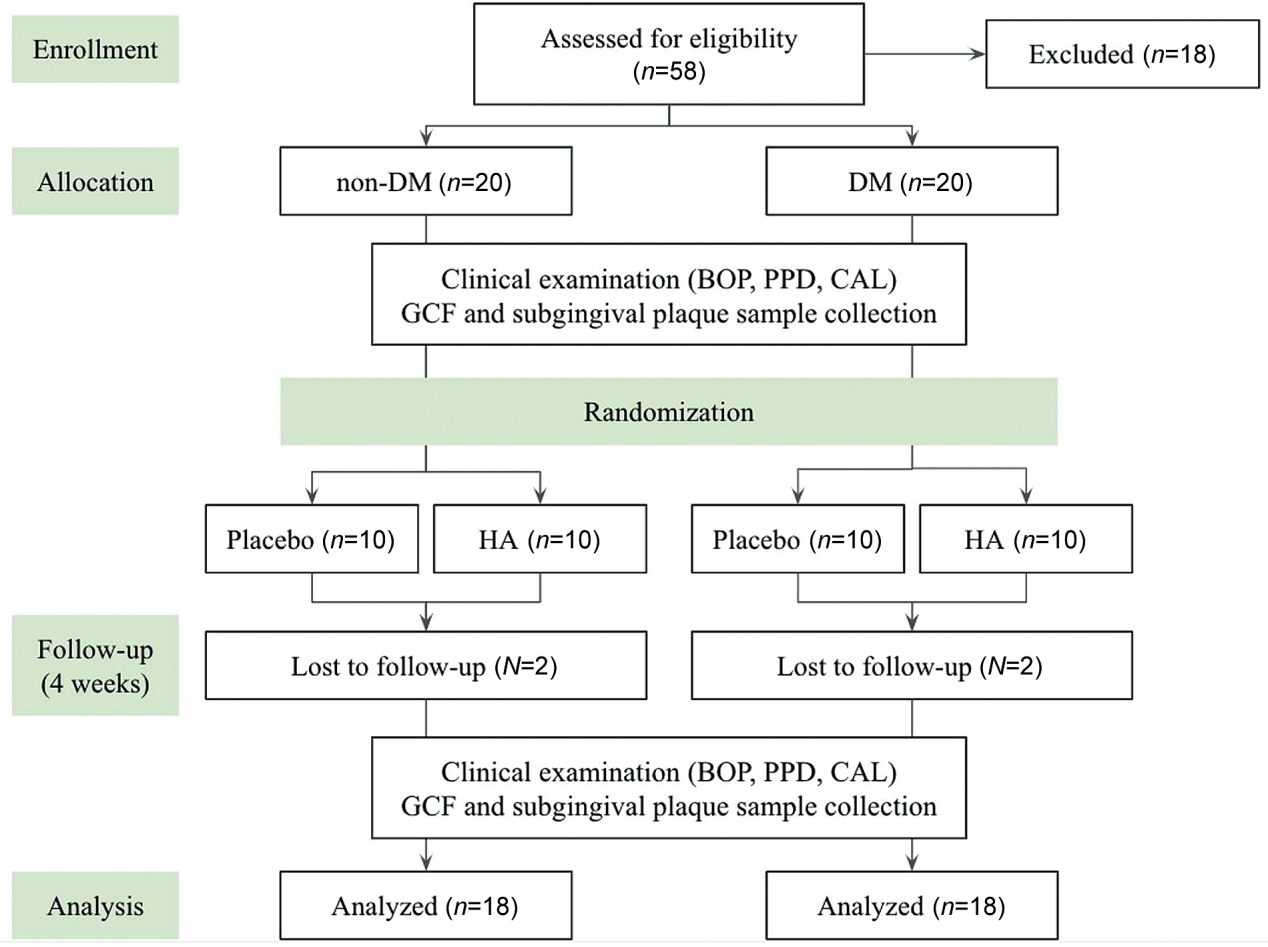
Flowchart of the study design. BOP, bleeding on probing; CAL, clinical attachment loss; DM, diabetes mellitus; GCF, gingival crevicular fluid; HA, hyaluronic acid; PPD, pocket probing depth.

This study was done in February until June 2023. Fig. 1 presents the flowchart of the study design. The study initiated with a total of 40 subjects, of which two healthy subjects and two diabetic subjects were lost to follow-up due to their inability to attend the specified follow-up appointment. A total of 36 selected patients completed the clinical trial. Patients include 23 females and 13 males, with a total of 18 patients in each group, DM (13 female and 5 male) and non-DM (10 females and 8 males). Each group is then divided equally to randomly receive different adjunctive gels after SRP. No adverse events were reported by all participants. Data in Table 2 reports general and diabetes-related parameters of the study groups.

**Table 2:** Baseline characteristics of patients

Groups	N	Age (years) mean ± SD	HbA1c (%) mean ± SD	Sex (M:F)
Non-DM, Placebo	9	50.78 ± 7.823	5.356 ± 0.6085	5: 4
Non-DM, HA gel	9	54.67 ± 6.827	5.467 ± 0.5831	3: 6
DM, placebo	9	47.89 ± 6.856	7.378 ± 0.7949	1: 8
DM, HA gel	9	57 ± 8.456	7.278 ± 0.6119	4: 5

Abbreviations: DM, diabetes mellitus; HA, hyaluronic acid; HbA1c, hemoglobin A1c; SD, standard deviation.

### Periodontal Clinical Parameters

Table 3 displays mean and standard deviation of BOP, PPD, and CAL at initial assessment (T _0_ ) and follow-up (T _1_ ) including differences between the two visits. Most of the groups demonstrated statistically significant decreases in all clinical parameters at the 4 weeks follow-up compared to the baseline ( *p* ≤ 0.05). Notably, the reductions in PPD and CAL were not statistically significant for the placebo ( *p* = 0.102 and *p* = 0.095 respectively) non-DM group. While significant improvements were observed at the follow-up compared to the baseline, only CAL exhibited a statistically significant intergroup ( *p* = 0.042) difference when comparing the changes between the two time points. Post-hoc results.

**Table 3:** Periodontal parameters at baseline and 4-week follow-up in DM and non-DM subjects

Periodontal parameters	Groups ( *n* = 9)		Mean ± SD		*p* -Value *	Δ change	
			Baseline (T _0_ )	4 weeks (T _1_ )		Mean ± SD	*p* -Value **
BOP (%)	Non-DM	Placebo	56.0 ± 17.84	26.22 ± 13.645	0.004 a*	29.78 ± 9.602	0.794 a
		HA gel	44.89 ± 18.811	20.22 ± 17.782	0.007 a*	24.67 ± 8.411	
	DM	Placebo	53.22 ± 30.298	27.67 ± 21.071	0.004 a*	25.56 ± 16.905	
		HA gel	63.56 ± 25.125	35.22 ± 19.344	0.002 a*	28.33 ± 12.062	
PPD (mm)	Non-DM	Placebo	4.56 ± 0.726	4.0 ± 1.0	0.102 b	0.56 ± 0.882	0.084 b
		HA gel	5.11 ± 0.782	3.44 ± 0.882	0.000 a*	1.67 ± 0.707	
	DM	Placebo	4.89 ± 0.928	4.0 ± 1.323	0.023 b*	0.89 ± 0.782	
		HA gel	4.78 ± 0.972	3.78 ± 1.641	0.037 b*	1.0 ± 1.118	
CAL (mm)	Non-DM	Placebo	5.11 ± 1.833	4.56 ± 2.186	0.095 a	0.56 ± 0.882	0.042 b*
		HA gel	5.0 ± 0.866	3.22 ± 0.972	0.006 b*	1.78 ± 0.667	
	DM	Placebo	6.11 ± 1.691	5.33 ± 2.291	0.023 a*	0.78 ± 0.833	
		HA gel	5.33 ± 2.398	4.33 ± 3.162	0.028 a*	1.0 ± 1.118	

Abbreviations: ANOVA, analysis of variance; BOP, bleeding on probing; CAL, clinical attachment loss; DM, diabetes mellitus; HA, hyaluronic acid; PPD, pocket probing depth; SD, standard deviation.

* Statistically significant p≤0.05 ^a^ Paired *t* -test or ^b^ Wilcoxon signed-rank test.

** Statistically significant p≤0.05 ^a^ One-way ANOVA test and posthoc Tukey or ^b^ Kruskal–Wallis test and posthoc Dunn-Bonferroni.

**Table 4:** IL-1β and IL-10 levels at baseline and 4-week follow-up in DM and non-DM subjects

Cytokine	Groups ( *n* = 9)		Mean ± SD		*p* -Value *	Δ change	
			Baseline (T _0_ )	4 weeks (T _1_ )		Mean (SD)	*p* -Value **
IL-1β (pg/mL)	Non-DM	Placebo	97.137 ± 17.024	72.562 ± 10.280	0.008 b*	24.575 ± 17.871	0.75 b
		HA gel	105.054 ± 12.168	79.444 ± 7.838	0.001 a*	25.610 ± 8.918	
	DM	Placebo	101.257 ± 20.961	77.918 ± 9.114	0.018 a*	23.339 ± 23.727	
		HA gel	102.992 ± 18.275	81.366 ± 20.629	0.021 b*	21.626 ± 22.317	
IL-10 (pg/mL)	Non-DM	Placebo	348.868 ± 58.141	587.152 ± 68.105	0.000 a*	−238.284 ± 82.518	0.212 a
		HA gel	407.446 ± 69.733	581.683 ± 59.746	0.001 a*	−174.238 ± 94.928	
	DM	Placebo	544.1 ± 96.719	704.831 ± 86.684	0.003 a*	−160.730 ± 112.717	
		HA gel	528.906 ± 137.957	639.879 ± 84.503	0.108 a	−110.974 ± 184.02	

Abbreviations: ANOVA, analysis of variance; DM, diabetes mellitus; HA, hyaluronic acid; IL-10, interleukin-10; SD, standard deviation.

* Statistically significant *p* ≤0.05 ^a^ Paired *t* -test or ^b^ Wilcoxon signed-rank test.

** Statistically significant *p* ≤0.05 ^a^ One-way ANOVA test and posthoc Tukey or ^b^ Kruskal–Wallis test and posthoc Dunn-Bonferroni.

Table 4 presents the levels of IL-1β and IL-10 cytokines in GCF at the baseline and follow-up. A statistically significant decrease in IL-1β is observed in all groups when comparing baseline to follow-up ( *p* ≤ 0.05). In the case of IL-10, a statistically significant increase is noted in all groups except for the DM HA gel group ( *p* = 0.108). Intergroup analysis reveals no significant differences ( *p* ≤ 0.05) for both proinflammatory and anti-inflammatory cytokines.

### Microbiological Monitoring

The findings reveal a reduction in the *Pg* bacteria proportion in both the DM and non-DM groups over the 4-week follow-up period, regardless of whether they received HA gel or placebo. Notably, the non-DM and DM placebo group showed significant test results in the 4 weeks ( *p* ≤0.05). In the DM HA group, which experienced an increase in the *Pg* bacteria proportion during the 4-week follow-up period. However, it is important to note that the results did not exhibit statistical significance in any of the groups ( *p* *≥* 0.05). Refer to Table 5 for more details.

**Table 5:** Microbial plaque relative expression at baseline and 4-week follow-up in DM and non-DM subjects

Microbial plaque relative expression	Groups ( *n* = 9)		Mean ± SD		*p* -Value *
			Baseline (T _0_ )	4 weeks (T _1_ )	
***Pg***	Non-DM	Placebo	1.00 ± 0.78	0.20 ± 0.21	0.005 a*
		HA gel	1.00 ± 1.55	0.42 ± 0.54	0.214 b
	DM	Placebo	1.00 ± 1.45	0.50 ± 1.17	0.028 b*
		HA gel	1.00 ± 1.63	2.07 ± 2.81	0.260 b
***Fn***	Non-DM	Placebo	1.00 ± 0.81	0.93 ± 1.00	0.441 b
		HA gel	1.00 ± 0.47	0.76 ± 0.59	0.395 a
	DM	Placebo	1.00 ± 1.31	0.76 ± 0.96	0.086 b
		HA gel	1.00 ± 0.70	0.99 ± 1.33	0.767 b

Abbreviations: DM, diabetes mellitus; Fn, *Fusobacterium nucleatum* ; HA, hyaluronic acid; IL-10, interleukin-10; *Pg* , *Porphyromonas gingivalis* ; SD, standard deviation.

* Statistically significant *p* ≤0.05 ^a^ Paired *t* -test or ^b^ Wilcoxon signed-rank test.

Furthermore, Table 5 also illustrates a decrease in the *Fn* bacteria proportion within both the DM and non-DM groups throughout the 4-week follow-up period, whether they received HA or a placebo, with nonsignificant results. Find additional details in Table 5 .

## Discussion

This current controlled, randomized, double-blind clinical study examines the impact of HA gel as adjunctive therapy to SRP in periodontitis patients diagnosed with or without DM through clinical, immunological and microbiological assessment. Our results revealed inconsistency, which may suggest a lack of significant benefits from the application of 0.2% HA gel to both healthy and diabetic patients.

Numerous clinical studies have explored the use of HA gel as an adjunctive therapy to SRP, which remains the established gold standard treatment for periodontitis. 30 However, to the best of the authors' knowledge, only one study has compared the efficacy of HA gel in patients with and without DM with periodontitis. Madkour et al presented findings related to periodontal clinical parameters, distinguishing this study as the first to examine immunological and microbiological parameters. 31

The average ages of subjects in the control groups for non-DM and DM categories are notably higher, with values of 50.78 ± 7.823 years and 47.89 ± 6.856 years, respectively, compared to those in the respective test groups, which have average ages of 54.67 ± 6.8273 years and 57 ± 8.456 years. Notably, there is an approximate 10-year difference in age between the control and test groups of DM subjects, which could potentially lead to variations in oral hygiene practices. 32 Additionally, an imbalance in the gender ratio was observed, with 63.89% being female. However, this gender imbalance may not significantly impact the study's outcomes, as hormonal changes in women primarily influence the flow of GCF than its composition, 33 thereby potentially causing no bias.

Our findings have demonstrated noticeable improvements in all clinical parameters at the follow-up compared to the baseline. However, in this case, the use of additional HA gel did not demonstrate a clear advantage, as there was no statistically significant difference ( *p* > 0.05) in the reduction in BOP and PPD between the two treatment groups. The sole statistically significant ( *p* ≤ 0.05) difference was observed in CAL gain among non-DM patients in both treatment groups. The result of several studies of adjunctive HA gel in non-DM patients varies most suggests that the superiority of HA gel may not be evident across all clinical parameters universally, some demonstrate significant changes in PPD, others in CAL, and yet others in BOP. 34 35 36 37 38 39 The use of HA as an adjunctive treatment alongside SRP has been under investigation for more than two decades, to the extent that a systematic review has been published on this subject. A systematic review and meta-analysis by Eliezer et al reported results in CAL gain, PPD and BOP reduction favoring nonsurgical therapy with HA. However, it is important to note that the author highlights high heterogeneity among the included studies that could be attributed to variations in treatment protocol, such as differences in HA gel concentration, the optimal timing for HA application, and the frequency of HA gel application. 40

The literature has well-established the anti-inflammatory, antiedematous, and antibacterial properties of HA. 41 42 43 In periodontitis, where infrabony defects may be present, hyaluronan undergoes degradation by reactive oxygen species primarily generated by infiltrating polymorphonuclear leukocytes and other inflammatory cells during the phagocytosis of bacteria. 44 After periodontal therapy, application of HA plays an indirect role in mitigating inflammation and stabilizing granulation tissue by inhibiting enzymes, such as serine proteinases, derived from inflammatory cells. These enzymes are responsible for the degradation of extracellular matrix proteins, and their inhibition contributes to the maintenance and stability of tissue during the healing process. 37 In infrabony defects, HA has the ability to accelerate bone regeneration through chemotaxis, proliferation, and differentiation of mesenchymal cells. HA also shares bone-inductive characteristics with osteogenic substances such as bone morphogenetic protein-2 and osteopontin. 45 Most clinical studies have primarily concentrated on clinical periodontal parameters. Research into the impact of HA on proinflammatory and anti-inflammatory cytokines as well as microbiological parameters remains limited.

In terms of cytokine levels, our study has identified a consistent pattern of reduced proinflammatory cytokine IL-1β and increased anti-inflammatory cytokine IL-10. The alterations in cytokine levels between baseline and follow-up prove statistically significant, with the exception of IL-10 in the DM HA group. However, when comparing the differences across all four groups, statistical significance is not observed for either of the cytokines. As a result, it becomes challenging to establish the superiority of 0.2% HA gel, and SRP alone appears to be sufficiently effective in treating periodontitis. The observed variability is likely attributed to the diverse concentrations and compositions of commercially available HA gels. The specific HA gel used in our study featured a native 0.2% HA gel composition. Notably, native HA is characterized by a relatively brief half-life, typically spanning 12 hours up to 3 days. 46 Cross-linked HA is recognized for its enhanced mechanical properties, suggesting that it might present a more viable option for future studies due to its extended duration compared to native HA. 47

Our observations regarding the proinflammatory cytokines differ from those in a study conducted by Mohammad et al, which reports a substantial decrease in the proinflammatory cytokines IL-1β and TNF-α after 6 weeks when comparing SRP with and without 0.8% HA gel. 36 Regarding anti-inflammatory cytokines, we have not come across a clinical study that focuses on the evaluation of IL-10 specifically in the context of HA treatment for periodontitis. Additional research on periodontitis has documented alterations in various inflammatory biomarkers, including an upregulation of endothelin-1, transforming growth factor-beta 1, and vascular endothelial growth factor. 48 49

In this study, it is evident that there is a connection between the presence of bacteria and changes in cytokines within the periodontal tissues. The presence of *Pg* and *Fn* leads to an elevation in the levels of IL-1β and several other proinflammatory cytokines. 50 51 52 53 To counterbalance this proinflammatory response, IL-10 levels also increase to downregulate inflammatory response in presence of bacteria. However, following periodontal treatment, a reduction in the bacterial load occurs, resulting in a decline in proinflammatory cytokines, which is indicative of decreased inflammation. Simultaneously, anti-inflammatory cytokines work to suppress inflammation and facilitate the healing process. 54 55 56

As far as the researchers are aware, most randomized controlled trials that concentrate on periodontal treatment in diabetic patients primarily aim to compare various treatment approaches and evaluate clinical parameters and enhancements in glycemic control. 57 58 59 60 Additionally, there is consistent evidence supporting the concept that nonsurgical periodontal therapy has a discernible positive effect on HbA1c levels in individuals with type 2 DM and chronic periodontitis.

Despite local changes in the periodontium among patients with type 2 DM, marked by heightened production of reactive oxygen species and proinflammatory cytokines such as IL-1, IL-6, and TNF-α, 61 and a higher microbial load, including *Pg* , 62 Christgau et al 63 and Taşdemir et al. 58 observed that well-managed diabetic individuals may respond to nonsurgical periodontal therapy as effectively as patients without type 2 DM who are in good health. In a study that also involved the analysis of bacteria, de Cruz et al 64 found no notable discrepancy in clinical and laboratory parameters between individuals with and without type 2 DM who had generalized chronic periodontal disease 3 months after undergoing SRP therapy.

Our study focuses on one red complex bacteria, *Pg* , which is also known as the keystone pathogen in periodontitis 65 along with one orange complex bacteria, *Fn* . 66 A decrease in both bacteria is seen in almost all groups, with a significant reduction found in *Pg* in the non-DM and DM placebo groups. Interestingly, the DM HA group exhibits an increase in the proportion of *Pg,* although this increase is not statistically significant. We speculate that the observed outcome could be linked to the approximately 10-year age difference between the test and control groups of subjects with type 2 DM, potentially resulting in decrease in oral hygiene maintenance. Parthasarathy et al reported a statistically significant increase in the percentage of poor plaque scores among individuals aged over 50 compared to those in the 35 to 50 years age group. 32

While *in vitro* studies have shown that HA significantly impedes the growth and formation of biofilms by *Pg* , 67 68 clinical investigations have not yet established the effectiveness of HA in reducing bacteria. In a systematic review conducted by Alshehri and Alharbi, 69 it was concluded that incorporating HA as an adjunct to nonsurgical mechanical therapy for periodontitis did not provide an additional benefit in reducing the presence of *Pg* in subgingival biofilms. Nguyen et al presented varying outcomes for different bacteria. *Porphyromonas gingivalis* exhibited a statistically significant decrease at the 6-week follow-up, whether HA gel was used or not. In contrast, *Fn* showed a significant reduction only when HA gel was included. However, when comparing the reduction in both treatment groups, there was no statistically significant difference between treatment with or without HA gel. It was noted that the disparity in results could be attributed to the specific location of the periodontitis and the collection of subgingival plaque from the deepest sites, which may not have provided a fully representative sample. One possible explanation could be that HA is potentially more effective in moderate periodontal pockets rather than deep ones. 39

In general, we observed a pattern that is consistent with most research outcomes, demonstrating enhancements in clinical parameters, reductions in proinflammatory cytokines and *Fn* bacteria, as well as an increase in anti-inflammatory cytokines across all study groups, though these changes are not statistically significant. The only parameter that did not exhibit a clear trend was the quantity of *Pg* . In addition to the treatment provided, the adherence of patients to oral hygiene instructions is a crucial factor in the effectiveness of periodontal treatment. 70 The application of HA in both DM and non-DM patients did not yield superior treatment outcomes when compared to groups receiving a placebo, which contradicts the findings from some *in vitro* studies. 41 67 The observed patterns indicate the effectiveness of periodontal treatment, as evidenced by reduced inflammation and improved clinical parameters of periodontal health. Enhancing periodontal tissue health is associated with lower HbA1c test results, translating to an improved overall quality of life for patients. This further substantiates the connection between type 2 DM and gum disease, underscoring the crucial role of incorporating gum treatment into diabetes prevention strategies. 60

The protocol for administering HA gel remains uncertain, as different studies have employed varying approaches, including differences in the concentration, formulation, frequency, and timing of HA application. The diverse protocols and concentrations of AH gel in the literature lead to heightened variability and inconsistency. We utilized 0.2% HA gel due to the unavailability of alternative concentrations. As we look ahead to future investigations, it is advisable to explore the potential benefits of higher HA concentrations. This exploration should extend beyond the examination of clinical parameters and encompass an evaluation of biomolecular and microbiological aspects. Consequently, further research is warranted to establish a standardized formula and protocol for achieving the full efficacy of HA as an adjuvant to SRP in clinical applications for both diabetic and non-diabetic patients.

This study places a strong emphasis on external validity, emphasizing the generalizability of our findings beyond the specific study conditions. We achieve this by thoughtfully selecting subjects that represent a broader population of interest, with a particular focus on individuals with DM. The study design is carefully developed, taking into consideration its potential impact on the community and the practical implications extending beyond research participants. These intentional efforts are aimed at ensuring that our findings not only enrich existing knowledge but also have meaningful applications in diverse settings, thereby enhancing the external validity of our research.

This study is subject to several limitations that require consideration. First, the inherent variability among individuals may introduce discrepancies in the obtained results, emphasizing the need for cautious interpretation. Second, our analysis centered on PPD from a singular site per participant, potentially limiting the holistic understanding of the observed conditions. Third, the unavailability of HA gel in higher concentrations posed a constraint on exploring potential dose-dependent effects. Lastly, the study's scope is circumscribed by a restricted number of follow-up assessments and a relatively modest sample size, raising awareness about the limitations in generalizing findings to the broader population.

## Conclusion

Adjunctive use of 0.2% HA gel to periodontal pockets alongside SRP did not provide significant additional benefits compared to SRP alone for both diabetic and healthy individuals in clinical, biomolecular, and microbiological parameters ( *p* > 0.05). While previous reports highlight the effective support of HA in periodontal wound healing, the study underscores the need to establish a well-defined protocol for the adjunctive use of HA gel to realize significant advantages.
